# Physical Activity, Sedentary Behavior, and Sleep Quality in Adults with Primary Hypertension and Obesity before and after an Aerobic Exercise Program: EXERDIET-HTA Study

**DOI:** 10.3390/life10080153

**Published:** 2020-08-17

**Authors:** Aitor Martinez Aguirre-Betolaza, Iñigo Mujika, Paul Loprinzi, Pablo Corres, Ilargi Gorostegi-Anduaga, Sara Maldonado-Martín

**Affiliations:** 1 Department of Physical Education and Sport. Faculty of Education and Sport-Physical Activity and Sport Sciences Section, University of the Basque Country (UPV/EHU), 01007 Vitoria-Gasteiz. Araba/Álava, Basque Country, Spain; pablo.corres@ehu.eus (P.C.); ilargi.gorostegi@ehu.eus (I.G.-A.); sara.maldonado@ehu.eus (S.M.-M.); 2 GIzartea, Kirola eta Ariketa Fisikoa Ikerkuntza Taldea (GIKAFIT), Society, Sports, and Physical Exercise Research Group, University of the Basque Country (UPV/EHU), 01007 Vitoria-Gasteiz. Araba/Álava, Basque Country, Spain; inigo.mujika@inigomujika.com; 3 Department of Physiology, Faculty of Medicine and Nursing. University of the Basque Country (UPV/EHU), 48940 Leioa, Basque Country, Spain; 4 Exercise Science Laboratory, School of Kinesiology, Faculty of Medicine, Universidad Finis Terrae, 7501015 Santiago, Chile; 5 Department of Health, Exercise Science, and Recreation Management, The University of Mississippi, Oxford, MS 38677, USA; pdloprin@olemiss.edu

**Keywords:** physical inactivity, questionnaire, objective measure, sleep, supervised exercise

## Abstract

Background: The purposes of the study were to: analyze, by objective (accelerometry) and subjective (International Physical Activity Questionnaire, IPAQ) methodologies, the physical activity (PA) and sedentary behavior (SB) in healthy adults (HEALTHY, *n* = 30) and individuals with primary hypertension (HTN) and overweight/obesity (*n* = 218); assess the effects of an aerobic exercise intervention on physical activity (PA), sedentary behavior (SB), and sleep quality in the HTN group; and evaluate the relationship between objectively measured and subjectively reported PA and SB. Methods: The measurements were performed before a 16-week exercise intervention period in both HEALTHY and HTN groups and after the intervention period only in the HTN group, randomized to attention control or exercise training (ExT) subgroups. Results: The HEALTHY group showed more moderate-to-vigorous PA (*p* < 0.05) and better sleep quality (*p* < 0.05) than the HTN group, but no difference in SB. After the intervention, HTN participants’ PA and SB, objectively measured by accelerometry, were unchanged, but increased PA and decreased SB (*p* < 0.05) were observed through IPAQ in ExT. The intervention was effective in improving sleep quality in HTN participants. Conclusions: The differences in moderate-to-vigorous PA and SB may be useful in defining the health profile of a population. The supervised aerobic exercise program was effective in increasing PA, reducing SB, and improving sleep quality in overweight/obese adults with HTN. Accelerometer-measured and self-reported data were not comparable, but complementary.

## 1. Introduction

There is clear and unanimous evidence on the benefits of regular physical activity (PA) for several health conditions [1,2], but also on the deleterious effects associated with sedentary behavior (SB; i.e., any waking behavior characterized by an energy expenditure ≤ 1.5 metabolic equivalents, while in a sitting, reclining, or lying posture) and physical inactivity (i.e., when an individual does not meet the PA recommendations) [3].

Historically, intervention efforts to counter PA have focused on moderate-to-vigorous PA (MVPA) [4]. However, updated international PA and SB guidelines for adults are promoting not only 150–300 min per week of MVPA and strengthening exercises, but also to reduce and interrupt prolonged SB with episodes of light-intensity PA (LPA) [5,6,7].

In 2012, physical inactivity was characterized as a global pandemic [8], given that SB is associated with a higher risk of mortality. Over the past few years, attention has been directed toward an alternative approach consisting of frequent breaks in sedentary time to reduce SB and increase LPA. Light-intensity PA has been shown to be relevant for those who are not regularly physically active, and higher LPA relative to SB provides additional benefits to those meeting PA guidelines [3,9,10]. However, it seems that leisure-time MVPA may not be protective for those who spend large amounts of time in SB [11]. The basic premise is that sitting too much is not the same as lack of exercise and, as such, has its own unique metabolic consequences, due to the identification of unique mechanisms that are distinct from the biological bases of exercising [12]. After the pioneering work of Dr. Jerry Morris in the 1950s [13], numerous studies have shown that bouts of sitting time and lack of muscular movement (i.e., SB) are strongly associated with obesity, abnormal glucose metabolism, diabetes, metabolic syndrome, cardiovascular disease, and cancer [2,11,14]. This association is independent of MVPA [15,16] and SB is also an independent determinant of chronic disease and all-cause mortality [17]. This observation emphasizes the beneficial effects of breaks in sedentary time, even when the same total amount of MVPA is performed [18], suggesting that SB carries a unique and independent risk to health that may not be reduced simply by becoming more physically active [2].

There are multiple ways of analyzing PA levels and SB, but the high variability among different methods makes comparisons problematic. The International Physical Activity Questionnaire (IPAQ) measures time spent sitting and PA at different intensities with demonstrated validity and reliability [16], and an acceptable validity against accelerometers [19]. Accelerometers are indeed another reliable and objective method to measure daily PA with no reporting bias. However, some authors have recently highlighted the inability of some accelerometers to capture non-step based or water-based PA (e.g., cycling or swimming), resulting in an underestimation of the amount of PA performed [20]. Besides, the lack of agreement about where the device must be placed complicates the interpretation and comparison of results between studies [21].

Accelerometers are also a validated and practical way of capturing and analyzing sleep quality parameters [22] due to their minimal influence on natural sleep [23]. There is clear evidence to suggest that inadequate or poor sleep patterns have adverse effects on cardiovascular, endocrine and immune function, body composition, and risk of mortality [24,25,26]. It has also been demonstrated that inadequate sleep duration and poor sleep quality are associated with restricted PA, and their association is bidirectional [27]. Previous studies have shown that poor sleepers are less likely to meet PA guidelines and that better sleep quality predicted higher levels of PA [27]. Results generally suggest that increasing PA levels will improve sleep and SB [28], although some studies found little or no effect in reducing total SB time [29,30]. It is also unclear how variables such as exercise type, duration, and intensity affect SB and sleep quality.

This study aimed to analyze the interactions between aerobic exercise training, PA, SB, and sleep quality in overweight/obese adults with primary hypertension (HTN). The specific purposes of the present study were: (1) to analyze, by objective (accelerometry) and subjective (IPAQ questionnaire) methodologies, the PA and SB in healthy adults (HEALTHY group) and individuals suffering from HTN and overweight/obesity (HTN group); (2) to assess the effects of a 16-week aerobic exercise intervention on PA, SB, and sleep quality in a HTN population with overweight/obesity; and (3) to assess the relationship between objectively measured and subjectively reported PA and SB.

## 2. Materials and Methods

### 2.1. Study Design

The EXERDIET-HTA study is a multi-arm parallel, randomized, single-blind, controlled, experimental trial comparing the effects of different 16-week aerobic exercise programs (performed 2 days/week) in overweight/obese participants with HTN (www.clinicaltrials.gov, number NCT02283047). The study protocol was approved by the Ethics Committee of the University of the Basque Country (UPV/EHU, CEISH/279/2014) and clinical investigation unit of Araba University Hospital (2015–030). Medical staff were blinded to the participant randomization process. The design, selection criteria, and procedures for the EXERDIET-HTA study have been detailed previously [31].

### 2.2. Participants

Two hundred and eighteen non-Hispanic white participants (*n* = 138 men (63.3%) and *n* = 80 women (36.7%)) with a diagnosis of HTN according to European guidelines [32] or who were taking pharmacological treatment for HTN and who were overweight or obese (body mass index (BMI) ≥ 25 kg/m^2^) with a sedentary and physically inactive lifestyle (according to IPAQ and below the “*Global Recommendations on Physical Activity for Health*” set by the World Health Organization) [33] took part in the study. All participants provided written informed consent before any data collection. A sample of healthy individuals (HEALTHY, *n* = 30) was also recruited from the community (approved by the Ethics Committee of the University of the Basque Country, UPV/EHU, M10/2018/229) and excluded if they had any chronic medical illness, were taking any daily prescription medications, had current medical symptoms, had abnormal findings on physical examination (including blood pressure (BP) ≥ 140/90 mmHg, or overweight BMI ≥ 25 kg/m^2^), or had abnormal results on a cardiac screening test (resting and exercise electrocardiogram).

### 2.3. Measurements

The measurements for the study were performed before the 16-week exercise intervention period (T0) in both HEALTHY and HTN samples, and after the intervention period (T1) only in the HTN group.

In order to assess the PA and SB, participants completed the short-form IPAQ [34], and wore a triaxial accelerometer (ActiGraph GT3X+, Pensacola, FL, USA) on their non-dominant wrist with a Velcro strap for eight consecutive days at all times, except during water-based activities. Each participant was given oral instructions on how to wear the accelerometer and how to complete the diary log. On the eighth day after the accelerometers were distributed, both accelerometers and diaries were collected. Accelerometer data were downloaded, cleaned, and analyzed using the manufacturer’s software (Actilife 6 desktop). For the analysis of SB, the cut-off point was <1853 counts per minute [35]. We measured the total PA (TPA), with the cut-off point >1853 counts per minute, because wrist intensity cut-points for adults using ActiGraph accelerometers were not validated. For SB analysis, sleep data were not included and were analyzed separately.

Sleep measures were analyzed using a validated software algorithm based on the Cole–Kripke scoring method [36] and calculated from the raw accelerometer data for each unit at 60-s epoch length. The following sleep variables were obtained from accelerometer data: bedtime (total time spent in bed); total sleep time (TST, min of sleep between sleep onset and wake time); and sleep efficiency (the ratio between TST and total time spent in bed × 100), of which values below 85% are usually considered clinically significant [37].

All participants answered the STOP-Bang Questionnaire for the screening of obstructive sleep apnea (OSA). This tool consists of eight dichotomous (yes/no) items (snoring, tiredness, observed apnea, BP medication, BMI, age, neck circumference, and sex). The total score ranges from 0 to 8. Individuals can be classified for OSA risk based on their respective scores (i.e., low risk < 2, moderate range 3–4, and high risk > 5–8). Neck circumference was measured in the midway of the neck, between the mid-cervical spine and mid-anterior neck, with the participants standing upright [38].

### 2.4. Intervention

After baseline measurements at T0, HTN participants were randomly allocated to one of the intervention subgroups stratified by sex, systolic BP, BMI, and age using a time-blocked computerized randomization program. The four intervention groups were: attention control group (AC) and three supervised exercise groups (high-volume and moderate-intensity continuous training, high-volume and high-intensity interval training, and low-volume and high-intensity interval training). A preliminary analysis of the data used in this research showed that there were no differences in the target variables among the three supervised exercise groups. Therefore, for the purposes of this study, all three supervised exercise groups were pooled together in a single group called exercise training (ExT). Comparative analyses were performed between the two HTN subgroups (AC vs. ExT). All participants followed a hypocaloric DASH diet (Dietary Approaches to Stop Hypertension) [39]. Habitual food consumption and nutrient intake were evaluated using three questionnaires: Dietary History, Food Frequency Questionnaire, and 24 h Recall Questionnaire. Every two weeks, participants were weighed and received encouragement and advice alongside nutritional counselling in order to aid compliance. Moreover, all participants were given PA advice to meet the global PA recommendations.

The ExT subgroup trained two non-consecutive days per week under the supervision of exercise specialists. All sessions started and finished with BP monitoring, and training intensity was dictated by individual heart rate responses (Polar Electro, Kempele, Finland) and the rate of perceived exertion (RPE, Borg’s 6–20-point scale). Each session lasted for approximately one hour and included a 5–10-min warm-up and a 10-min cool-down. The main part of each training session consisted of a range of aerobic exercises: one day of the week on a treadmill (BH Fitness, Vitoria-Gasteiz, Basque Country) and the second day on a stationary bike (BH Fitness, Vitoria-Gasteiz, Basque Country). The intensity of the exercise was individually tailored by adjusting the speed and/or the incline of the treadmill, and the power output and/or the pedaling cadence on the exercise bike. Additional information on the supervised exercise training protocols can be found elsewhere [31].

### 2.5. Statistical Analysis

Descriptive statistics were calculated for all variables and presented as mean ± standard deviation (SD). Comparisons between the HEALTHY and HTN groups were performed with independent *t*-tests. Analysis of variance was used to determine if there were significant differences at baseline (T0) between the three groups of supervised exercise.

A related two-sample *t*-test was used to assess the effects of the intervention (T0 vs. T1) within each HTN subgroup (AC and ExT). Analysis of covariance was used to examine the delta (∆) score for each HTN subgroup and an independent two-sample *t*-test was performed to determine the differences between groups at T1.

Pearson correlation coefficients were used to assess the relationship between objectively measured and subjectively reported sedentary time and TPA. Weighted histograms were used to present the distribution of the mean difference between measured and reported SB (calculated as measured estimate – reported estimate) in the entire HTN sample before the intervention and the ExT subgroup after the intervention.

Data were analyzed according to the intention-to-treat principle. Statistical significance was set at *p* < 0.05. All statistical analyses were performed with Statistical Package for the Social Sciences (SPSS) version 24.0.

## 3. Results

Previous reports for the EXERDIET-HTA study have already presented the baseline body composition, BP, cardiorespiratory fitness, biochemical profile, and medication intake data of the HEALTHY and HTN groups [40], as well as changes in these variables elicited by the intervention in the HTN group [41,42,43].

**Figure 1:**
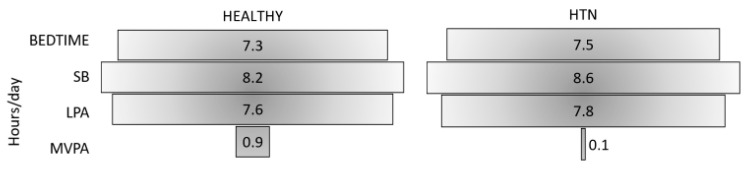
Accelerometer-measured and self-reported data for bedtime, sedentary behavior (SB), light-intensity physical activity (LPA), and moderate-to-vigorous physical activity (MVPA) in the healthy (HEALTHY) and primary hypertension (HTN) groups at baseline.

**Figure 2:**
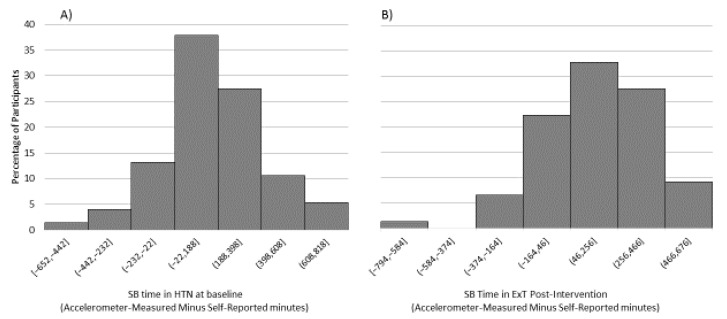
Distribution of the time difference between accelerometer-measured and self-reported sedentary behavior (SB) in (**A**) the primary hypertension (HTN) sample at baseline and (**B**) the exercise training subgroup (ExT) at post-intervention.

At baseline, HTN participants showed significantly lower levels of vigorous (*p* = 0.012) and moderate (*p* < 0.001) PA through IPAQ and of TPA (*p* = 0.042) measured by accelerometer compared with HEALTHY participants (Table 1, Figure 1). No differences (*p* > 0.05) between HEALTHY and HTN participants were found in SB irrespective of the method used. However, the weighted histograms showed that mean difference (accelerometer-measured minus self-reported) in SB time ranged between −22 and 188 min in 37% of HTN individuals (Figure 2). In addition, HEALTHY participants showed better sleep quality than HTN participants due to better sleep efficiency (*p* < 0.001) and longer sleep time (*p* = 0.017). Moreover, HTN individuals showed moderate risk for OSA, higher STOP-Bang Questionnaire scores (3.7 ± 1.3 vs. 0.6 ± 1.0, *p* < 0.001), and neck circumference (39.3 ± 3.9 vs. 33.6 ± 3.0 cm, *p* < 0.001) than HEALTHY individuals. No baseline differences were found in any variable between HTN subgroups.

After the intervention period (T1), PA levels did not increase (*p* > 0.05) and SB did not decrease (*p* > 0.05) with accelerometry measurements neither in AC nor in ExT subgroup (Table 2). On the other hand, IPAQ data indicated that the ExT subgroup significantly increased its level of vigorous PA (Δ = 51 min/week, 95% confidence interval (CI) = 68, 34 min/week) and moderate PA (Δ = 117 min/week, 95% CI = 157, 77 min/week), and decreased sitting time (Δ = −381 min/week, 95% CI = 94.2, −667 min/week). However, no significant differences between the subgroups (AC vs. ExT) were found. The mean difference (accelerometer-measured minus self-reported) in SB ranged between 46 and 256 min in 32.9% of ExT participants (Figure 2).

The sleep analysis showed (Table 3) that after the intervention both subgroups increased sleep efficiency (AC, Δ = 2.8%, 95% CI = 4.9, 8.2%; ExT, Δ = 4.4%, 95% CI = 5.7, 3.1%) and TST (AC, Δ = 18 min/day, 95% CI = 32, 4 min/day; ExT, Δ = 26 min/day, 95% CI = 37, 14 min/day) in the whole week, and also in the five weekdays: sleep efficiency (AC, Δ = 3.6%, 95% CI = 5.6, 1.5%; ExT, Δ = 4.5%, 95% CI = 5.8, 3.2%) and TST (AC, Δ = 23 min/day, 95% CI = 39, 7 min/day; ExT, Δ = 29 min/day, 95% CI = 42, 16 min/day). However, only the ExT subgroup increased its bedtime values (Δ = 13 min/day, 95% CI = 25, 1 min/day). During the weekend, only the ExT subgroup had improved sleep efficiency (Δ = 4.4%, 95% CI = 6.0, 2.8%). No statistical differences were apparent in the STOP-Bang Questionnaire scores after the intervention, but both subgroups decreased the total score, with a significant reduction in neck circumference (AC, Δ = −1.1 cm, 95% CI = −0.2, −1.9 cm; ExT, Δ = −0.8 cm, 95% CI = −0.4, −1.2 cm).

Pearson’s correlations between the accelerometer-measured variables and IPAQ were not significant neither for TPA (T0, *r* = 0.117, *P* = 0.117; T1, *r* = −0.106, *P* = 0.258; mean difference between pre- and post-values, *r* = −0.067, *P* = 0.502) nor for SB (T0, *r* = 0.132, *P* = 0.076; T1, *r* = −0.189, *P* = 0.054; *r* = 0.049, *P* = 0.624).

## 4. Discussion

To the best of our knowledge, this was the first study that analyzed the beneficial effect of an aerobic exercise program with nutritional intervention on SB, PA, and sleep variables in an overweight–obese population with HTN. The main findings of this study were: (1) the HEALTHY group showed more PA time and better sleep quality than HTN participants, with higher volume of MVPA, but no difference in SB time; (2) HTN individuals’ PA and SB times objectively measured by accelerometry were unchanged after the 16-week intervention, but increased MVPA and decreased SB times were observed through IPAQ in the ExT subgroup. IPAQ questionnaire and accelerometer results were thus not comparable; and (3) the intervention was effective in improving sleep quality in the HTN sample as a whole.

### 4.1. Baseline Results in HEALTHY and HTN

Under the premise that “too much sitting” is distinct from “too little exercise” [12], we could consider HEALTHY individuals of this study as sedentary (8.2 h/day in SB) but physically active (i.e., meeting current PA recommendations), while HTN participants were both sedentary (8.6 h/day in SB) and physically inactive. Arguably we are living in a sedentary society, regardless of meeting the current PA recommendations or not. Further, it seems clear that the risks associated with SB could be higher among people who are not regularly physically active [3], but also that a high level of MVPA might attenuate the adverse consequences of SB, as demonstrated by a harmonized meta-analysis of data from over 1 million men and women [44]. In the present study, the significant difference in MVPA level at baseline between HEALTHY (401 min/week or 0.9 h/day) and HTN (65 min/week or 0.1 h/day) individuals, and similar SB and LPA levels (Table 1, Figure 1) might be reflected in the initial unhealthy status of HTN [45]. Such findings highlight the close relation between physical inactivity and cardiovascular disease, and conversely the cardioprotective effect of regular MVPA secondary to high cardiorespiratory fitness [3]. Furthermore, the lack of differences in walking time (estimated by IPAQ, Table 1) and the percentage of TPA volume (measured by accelerometry, Table 1) between HEALTHY and HTN participants do not support the health-enhancing role of LPA showed by previous studies in older adults [9]. Therefore, LPA may not be sufficient to reduce cardiovascular risk factors in sedentary and physically inactive adults [17].

The subjective and objective benefits of regular MVPA on sleep are well-known [29], as is the strong association between short sleep duration, poor sleep quality, and cardiometabolic risk factors such as HTN and overweight/obesity [25,26]. Results of the present study seemed to confirm the aforementioned association. Although both groups spent similar time in bed (7.4–7.5 h/day, Table 1, Figure 1), the HTN group showed poorer sleep quality than the HEALTHY group due to significantly shorter sleep time per day (HEALTHY = 6.7 h vs. HTN = 6.4 h) and worse sleep efficiency (HEALTHY = 92% vs. HTN = 84%). Indeed, HTN participants did not reach 85% sleep efficiency, which is considered to be healthy [26,37,46]. The absence of MVPA and bad sleep quality, along with a moderate risk for OSA as shown by the STOP-Bang Questionnaire, could be the key factors explaining the unhealthy status of HTN participants, presenting with physical, clinical, and physiological differences compared with HEALTHY participants [40,42]. In this sense, our results were consistent with previous studies reporting that a high level of exercise was associated with reduced odds of moderate-severe OSA, which in turn was associated with increased all-cause mortality [38,47].

### 4.2. Intervention Effects in HTN Subgroups

Although the use of objective methods to assess PA and SB presents several advantages over questionnaires [48], some studies have pointed to the inability of accelerometers to capture water and non-step based PA, likely resulting in an underestimation of overall PA [20]. A recent investigation has even concluded that it is not possible nowadays to ascertain the prevalence of meeting the PA guidelines based on accelerometer data [21,49]. In this respect, no differences were found in the present study when PA and SB were compared before and after the 16-week intervention (T0 vs. T1) in either of the HTN subgroups. However, self-reported data from IPAQ indicated significant increases in MVPA and decreases in SB in the ExT subgroup. These results suggested that: (1) TPA may be underestimated and SB overestimated by accelerometry. This was due to non-step activities not being recorded (in this study, the participants exercised once a week on the bike and once a week on the treadmill) and wrist intensity cut-points for adults using ActiGraph accelerometers not being validated nor established [35,48]; (2) the weak correlations between accelerometer-measured and self-reported TPA and SB data in this study, both before and after the intervention, suggested that these methods are not to be used interchangeably, especially when non-step based PA is performed. Similarly, data from the weighted histograms (Figure 2) presented a high disagreement between measured and reported SB, with more than 200 min/day mismatch in every difference range of HTN participants at baseline, and a similar mismatch in the ExT subgroup after the intervention. These results were in agreement with previous studies analyzing the reliability and validity of IPAQ compared to accelerometer cut-off points in the quantification of SB and PA in older adults [50]. However, rather than simply comparing measured and estimated methods, the most effective strategy may be to benefit from the complementary information of both methods, as previously suggested [51].

After the intervention, the ExT subgroup markedly reduced the sitting time (∆ = −49.0%, from 6.7 to 3.4 h/day, *p* = 0.01) and increased MVPA, according to self-reported data (Table 2). These results, along with previously published analyses on the same sample showing improvements from unhealthy to healthy physical, clinical, and physiological profile [40,41,42]), further support the use of self-reported data from IPAQ questionnaire in parallel to accelerometer-measured data. Further, the benefits of increased PA and decreased SB were also observed after analyzing sleep quality, confirming that longer sleepers generally have better metabolic profiles [52]. Indeed, sleep efficiency improved (AC, ∆ = 2.8%; ExT, ∆ = 4.4%) and total sleep time increased (AC, ∆ = 17.9 min/day; ExT, ∆ = 37.3 min/day) in both subgroups after the intervention. Nevertheless, total sleep time and bedtime were higher only during weekdays in both subgroups, whereas on weekend days only ExT increased sleep efficiency (∆ = 4.4%). As expected, our results were in agreement with most studies showing that for healthier people, Sunday was the day with the highest levels of SB and sleep time, and lowest levels of MVPA, whereas frail individuals were consistently inactive every day of the week [20,53].

The current study showed evidence for the benefits of PA on reducing SB and improving PA levels and sleep quality, but some limitations should also be considered. Firstly, although the sample size was sufficient for the present study, it would not be comparable to that of larger epidemiological studies, and future studies should expand the sample size. Secondly, the AC subgroup’s daily PA could not be controlled, which could jeopardize the validity of the analyses in this group. Thirdly, the sample size imbalance among AC and ExT subgroups, which could have slightly distorted the results. As a strength, due to the lack of validated PA intensity cut-points for wrist-worn accelerometers, the combination of both objective and subjective methodologies to determine the intensities of daily activities made the present results more complete.

## 5. Conclusions

The present study showed that the differences in MVPA and SB may be useful in defining the health profile of a population. Further, a 16-week supervised aerobic exercise program was effective in increasing self-reported PA, reducing SB, and improving sleep quality in overweight/obese adults with HTN. Accelerometer-measured and self-reported data were not comparable, but they were complementary. The findings of this study highlight the need for a regular, scheduled, and supervised PA program to promote healthier habits in adults with HTN and overweight/obesity.

## Figures and Tables

**Table 1:** Physical activity and sleep parameters of the study sample at baseline.

				HTN SUBGROUPS		
	HEALTHY (*n* = 30)	HTN (*n* = 218)	*p*-Value HEALTHY vs. HTN	AC (*n* = 56)	ExT (*n* = 162)	*p*-Value AC vs. ExT
**IPAQ**						
VPA (min/week)	37 ± 52	10 ± 56	0.012 *	10 ± 40	10 ± 85	0.947
MPA (min/week)	364 ± 301	55 ± 125	<0.001 *	71 ± 129	50 ± 123	0.273
Walking (min/week)	223 ± 246	232 ± 249	0.846	238 ± 283	230 ± 237	0.838
Sitting (min/week)	2863 ± 1116	2908 ± 1387	0.865	3159 ± 1594	2821 ± 1301	0.120
**ACCELEROMETER**						
***Physical activity***						
Sedentary time (min/day)	494 ± 84	519 ± 125	0.295	508 ± 136	525 ± 124	0.438
Sedentary (%)	51.5 ± 8.5	54.6 ± 10.4	0.091	52.7 ± 9.5	51.1 ± 10.3	0.569
TPA (min/day)	478 ± 90	435 ± 108	0.042 *	460 ± 107	424 ± 109	0.056
TPA (%)	48.5 ± 8.5	45.4 ± 10.4	0.078	47.3 ± 9.5	48.9 ± 10.3	0.571
***Sleep***						
Efficiency (%)	91.7 ± 3.2	84.1 ± 7.0	<0.001 *	85.0 ± 5.7	83.5 ± 7.5	0.216
Bedtime (min/day)	442 ± 43	452 ± 63	0.287	445 ± 62	455 ± 64	0.372
TST (min/day)	405 ± 44	382 ± 70	0.017 *	380 ± 65	382 ± 73	0.865
STOP-Bang score	0.6 ± 1.0	3.7 ± 1.3	<0.001 *	3.9 ± 1.1	3.6 ± 1.5	0.171
Neck circumference (cm)	33.6 ± 3.0	39.3 ± 3.9	<0.001 *	39.5 ± 3.6	39.2 ± 4.1	0.841

Values are mean ± SD. HEALTHY, healthy group; HTN, primary hypertension group; AC, attention control subgroup; ExT, exercise training subgroup; IPAQ, International Physical Activity Questionnaire—short form; VPA, vigorous physical activity; MPA, moderate physical activity; TPA, total physical activity; efficiency (%), total sleep time divided by total bedtime multiplied by 100; TST, total sleep time; STOP-Bang score, points obtained in the STOP-Bang Questionnaire. * *p* < 0.05.

**Table 2:** Physical activity levels before (T0) and after (T1) 16 weeks of exercise intervention in the HTN subgroups.

	AC (*n* = 37)	*P*_T0-T1_	ExT (*n* = 109)	*P*_T0-T1_	*p*-Value AC vs. ExT
***Physical activity***					
**ACCELEROMETER**					
Sedentary time (min/day)					
T0	498 ± 105		544 ± 125		
T1	504 ± 98	0.788	519 ± 110	0.215	0.374
Sedentary time (%)					
T0	52.7 ± 10.4		55.8 ± 8.9		
T1	54.3 ± 10.0	0.496	54.2 ± 9.7	0.276	0.250
TPA (min/day)					
T0	459 ± 110		435 ± 92		
T1	434 ± 94	0.265	445 ± 99	0.448	0.172
TPA (%)					
T0	47.3 ± 10.4		44.2 ± 8.9		
T1	45.7 ± 10.0	0.496	45.8 ± 9.7	0.276	0.250
**IPAQ**					
VPA (min/week)					
T0	11 ± 43		11 ± 88		
T1	40 ± 101	0.080	62 ± 94	<0.001 *	0.221
MPA (min/week)					
T0	76 ± 135		47 ± 124		
T1	150 ± 204	0.056	163 ± 200	<0.001 *	0.308
Walking (min/week)					
T0	257 ± 292		237 ± 240		
T1	337 ± 397	0.286	288 ± 279	0.098	0.679
Sitting (min/week)					
T0	3200 ± 1566		2818 ± 1302		
T1	2636 ± 1146	0.060	1437 ± 1336	0.010 *	0.546

TPA, total physical activity; IPAQ, International Physical Activity Questionnaire—short form; VPA, vigorous physical activity; MPA, moderate physical activity; HTN, primary hypertension group; AC, attention control subgroup; ExT, exercise training subgroup. * *p* < 0.05.

**Table 3:** Sleep quality analysis before (T0) and after (T1) 16 weeks of exercise intervention in the HTN subgroups.

	AC (*n* = 37)	*P*_T0-T1_	ExT (*n* = 109)	*P*_T0-T1_	*p*-Value AC vs. ExT
***Sleep***					
*Complete week (7 days)*					
Efficiency (%)					
T0	84.8 ± 5.7		83.0 ± 8.0		
T1	87.6 ± 6.7	0.007 *	87.5 ± 4.9	<0.001 *	0.218
Bedtime (min/day)					
T0	441 ± 59		452 ± 60		
T1	449 ± 49	0.287	461 ± 62	0.128	0.978
TST (min/day)					
T0	375 ± 62		378 ± 70		
T1	393 ± 55	0.013 *	403 ± 60	<0.001 *	0.403
*Weekdays (5 days)*					
Efficiency (%)					
T0	84.3 ± 6.0		82.9 ± 7.9		
T1	87.9 ± 6.7	0.001 *	87.3 ± 4.8	<0.001 *	0.481
Bedtime (min/day)					
T0	431 ± 61		441 ± 64		
T1	442 ± 52	0.245	454 ± 72	0.041 *	0.897
TST (min/day)					
T0	365 ± 63		367 ± 72		
T1	388 ± 55	0.005 *	396 ± 67	<0.001 *	0.633
*Weekend (2 days)*					
Efficiency (%)					
T0	85.5 ± 6.4		83.3 ± 9.0		
T1	87.4 ± 7.4	0.160	87.8 ± 6.7	<0.001 *	0.123
Bedtime (min/day)					
T0	469 ± 75		478 ± 83		
T1	462 ± 75	0.556	475 ± 75	0.722	0.818
TST (min)					
T0	402 ± 77		401 ± 70		
T1	404 ± 73	0.874	417 ± 78	0.076	0.318
STOP-Bang score					
T0	3.7 ± 1.1		3.7 ± 1.5		
T1	3.2 ± 1.2	0.170	3.5 ± 1.2	0.280	0.140
Neck circumference (cm)					
T0	39.3 ± 3.6		39.4 ± 4.0		
T1	38.2 ± 3.0	0.015	38.6 ± 3.8	<0.001 *	0.536

Efficiency (%), total sleep time divided by total bedtime multiplied by 100; TST, total sleep time; STOP-Bang score, points obtained in the STOP-Bang Questionnaire; HTN, primary hypertension group; AC, attention control subgroup; ExT, exercise training subgroup. * *p* < 0.05.
